# Recognizing the new disorder “idiopathic hypocryoglobulinaemia” in patients with previously unidentified clinical conditions

**DOI:** 10.1038/s41598-022-18427-x

**Published:** 2022-09-01

**Authors:** Dario Roccatello, Savino Sciascia, Carla Naretto, Antonella Barreca, Laura Solfietti, Laura Battaglia, Lucia Viziello, Roberta Fenoglio, Daniela Rossi

**Affiliations:** 1 CMID-Nephrology and Dialysis Unit (ERK-Net, ERN-ReConnet, RITA-ERN Member), Research Center of Immunopathology and Coordinating Center of the Network of Rare Disease of Piedmont and Aosta Valley, S. Giovanni Bosco Hub Hospital and Department of Clinical and Biological Sciences, University of Turin, Piazza del Donatore di Sangue 3, 10154 Turin, Italy; 2 Pathology Division, Città della Salute e Della Scienza, Torino, Italy

**Keywords:** Autoimmunity, Health care, Rheumatology, Nephrology, Glomerular diseases, Membranoproliferative glomerulonephritis

## Abstract

A considerable number of patients with high clinical suspicion for cryoglobulinaemic vasculitis either show negative results for the detection of cryoglobulins or show only trace amounts which cannot be characterized for composition. We aimed at establishing whether the failure to detect or the detection of trace amounts of cryoglobulin with conventional methods either identifies a peculiar subset of low level cryoglobulinaemia (from now on *hypocryoglobulinaemia*) or represents a separate entity. Using a modified precipitation technique in hypo-ionic medium, we prospectively identified between 2008 and 2021 237 patients (median age 60.8 years [22–97], 137 females) having < 0.5% cryocrit and clinical suspicion of autoimmune disorder. Of these 237 patients, only 54 (22.7%) had a history of HCV infection. One hundred and sixty-nine out of 237 patients (71%) had an established underlying disease, while 68 patients (28.6%) (median age 62.9 years [29–93], 35 females) did not show either laboratory markers or clinical symptoms consonant with an underlying aetiology. These 68 cases with only trace amounts of cryoglobulins were defined as having a *putatively idiopathic* hypocryoglobulinaemia. Nineteen of these 68 patients (27.9%) had a history of HCV infection. Twenty-four patients out of 68 (35.3%) were positive for rheumatoid factor (RF), while 25 (36.7%) patients had signs of complement consumption (i.e., C4 < 15 mg/dl and/or C3 < 80 mg/dl ), and 36 (52.9%) had increased inflammatory indexes. Seven patients only had arthralgia and constitutional symptoms while 61 out of 68 (89.7%) presented with at least one of the three cardinal signs of cryoglobulinaemic vasculitis including skin lesions, peripheral nerve involvement, and glomerulonephritis. Seventy-five percent of the subjects had type III hypocryoglobulins. In patients with hypocryoglobulinaemia the histologic features of glomerulonephritis (also examined by electron microscopy) resembled those of mixed cryoglobulinaemia-associated glomerulonephritis. In conclusion, hypocryoglobulins are often polyclonal and are mainly unrelated to HCV infection. Patients who present high clinical suspicion for vasculitis, especially glomerulonephritis and yet test negative for cryoglobulinaemia detected by standard techniques, could require deeper investigation even in the absence of HCV infection, RF activity or signs of complement consumption.

## Introduction

Cryoglobulinaemia is an immune disorder caused by the presence in the circulation of cold-precipitable immunoglobulins^1–3^. Mixed cryoglobulinaemia (MC) is a combination of monoclonal IgM and polyclonal IgG (type II), or of polyclonal IgG and IgM (type Ill). A so-called type II–III mixed cryoglobulinaemia has also been identified and is characterised by an intermediate restriction stage from type III to II MC^4^. Both polyclonal and monoclonal IgM present with rheumatoid factor (RF) activity. Type III MC is more frequently identified in subjects with infectious or autoimmune conditions. Type II and type II–III, and sometimes type III MC, can be considered a separate clinical entity within the spectrum of the systemic vasculitis involving small and, more rarely, medium-sized vessels. Most of these cases are associated with the presence of a hepatitis C virus (HCV) infection^5,6^. Type II cryoglobulinaemia can also be identified in patients with low-grade proliferative B-cell lymphomas^7^. The incidence of cryoglobulinaemia varies across the globe^8^, with most of the cases described in France, Spain, Italy, and Israel^9^. Diagnosis may be either delayed or overlooked. Biomarkers of HCV infection can be detected in the majority of cases. The cryoprecipitate can present with HCV concentrations up to 1000 times higher than the serum supernatant^10^. While circulating cryoglobulins (asymptomatic in most of the cases) can be detected in about half of the subjects with chronic HCV^9^, a cryoglobulinaemic syndrome is observed in 2% of cases. Older age and longer HCV infection have been identified as predisposing factors for the development of cryoglobulinaemic vasculitis. Genetic factors can also play a role, with DRB1*11 more frequently observed in patients with kidney involvement, while DRB1*15 observed to be protective^11^.

The clinical presentation of MC varies from mild form (fatigue, arthralgias and mild palpable purpura) to aggressive vasculitic manifestations involving the skin (to include necrotic lesions), gastrointestinal tract, lungs, as well as the nervous system with both peripheral and, more rarely, central. Renal involvement is not infrequent and represents a main prognostic factor. It consists of a unique kind of nephropathy which has been found to be strongly associated with HCV infection^11^. The heterogeneity of histological features (potentially requiring different therapeutic strategies) makes the renal biopsy mandatory for the diagnosis unless specifically contraindicated in all subjects with cryoglobulinemia and unexplained renal impairment or urinary abnormalities. Electron microscopy is often resolutive, allowing the detection of characteristic electron-dense structured deposits in unclear cases.

Low values of C3, C4, and C1q, and slightly deranged to very high IgM RF can be detected along with type II cryoglobulins (IgM–k, polyclonal IgG). Very low, or undetectable, levels of C4 are often considered a very suggestive indicator of the presence of cryoglobulinaemia^12^. As cryoglobulins precipitate if the test sample reaches temperatures < 37 °C, extreme pre-analytic care is required to reduce the rate of false-negative results that lead to misinterpretation, such as seronegative MC disease^13^. Similarly, inappropriate sample management can cause the identification of only scanty amount of processable material. This is why a technique of precipitation in hypo-ionic medium has been adopted in our Centre since 2008 in order to detect even trace amounts of cryoglobulins.

The present study aimed at establishing whether the detection of trace amounts of cryoglobulin (from now on called *hypocryoglobulin*) either identifies a subset of the general disorder of cryoglobulinaemia (including the HCV-associated form) or represents a separate entity with peculiar features of clinical presentation.

## Results

Among the 237 patients with trace amounts of cryoglobulins (cryocrit < 0.5%), namely hypocryoglobulins, 54 (22.7%) had a history of HCV infection (present or eradicated), 177 (74.6%) had no HCV infection and 6 remained undetected. One hundred and sixty-nine out of 231 patients (73%) had an established underlying disease, mainly Rheumatoid arthritis, Primary Sjogren syndrome, Systemic lupus erythematosus, and Systemic Sclerosis. Upon careful evaluation, 68 patients (28.6%) (35 females and 33 males, mean age 62.9 years, range 29–93 years) did not show clinical symptoms consistent with an underlying aetiology as ruled out after multidisciplinary evaluation by infectious disease specialists, immunologists, nephrologists and rheumatologists. These cases were defined as having a *putatively idiopathic* hypocryoglobulinaemia. A comprehensive panel of serological and imaging investigations were performed in line with the clinical presentation. Nineteen out of 68 patients (27.9%) had a history of HCV infection. Genotype was determined in 10: five had genotype 1, three had genotype 2, one had genotype 3 and one had genotype 4. Twenty-four patients out of 68 (3.35%) were positive for RF, 25 patients (36.7%) had signs of complement consumption (C4 < 15 mg/dL and or C3 < 80 mg/dL), and 36 (52.9%) had increased inflammatory indexes (erythrocyte sedimentation rate and/or C-Reactive Protein). Type III hypocryoglobulins could be detected in 52 (76.4%) of the cases, and type II in 16 (23.5%).

Seven patients had only arthralgia and constitutional symptoms, while 61 (89.7%) out of 68 presented with at least one of the three cardinal signs of cryoglobulinaemic vasculitis, including: *skin lesions* (orthostatic purpura (# 34 including 22 cases with biopsy-proven leukocytoclastic vasculitis); *peripheral nerve involvement* as detected by electrophysiologic examination (# 28, in 14 cases associated with skin lesions); biopsy-proven *glomerulonephritis* (# 9). With regard to patients with glomerulonephritis, one presented with all 3 cardinal clinical manifestations (involving skin, peripheral nervous system and glomerulonephritis). In the other 8 cases glomerulonephritis was the preeminent manifestation of the disease. Two of them had transient arthralgia and 4 fleeting purpura. Two out of 9 patients were HCV positive, genotype 1b (with RNA copies 539.265 IU/ml and 1.593.118 IU/ml, respectively). The clinical presentations of the 9 patients with hypocryoglobulinaemia and biopsy-proven nephritis consisted of proteinuria of nephrotic range (> 3.5 g/24 h, range 3.5–10 g/24 h) in 4 patients and < 3.0 g/24 h (range 0.5 g–2.7 g/24 h) in the remaining 5, and haematuria in 5 patients (mean 91 RBC/HPMF, range 31–250). Serum creatinine was 1.0 mg/dL in one patient, and > 1.5 mg/dL (range 1.3–4.3 mg/dL) in 8. When comparing these patients defined as having idiopathic hypocryoglobulinaemia with patients of a historic cohort of mixed cryoglobulinaemia-associated nephritis described by our group^14^, we found a significantly lower prevalence of HCV+ (27% vs. 87%, p < 0.001), RF+ (35% vs. 97%, p < 0.001), and C4 consumption (37% vs. 97%, p < 0.001) in the group with idiopathic hypocryoglobulinaemia. Table 1 shows the comparison of patients with biopsy-proven renal involvement in the two groups, with multisystemic involvement and HCV+ being more frequent in the cohort of patients with mixed cryoglobulinaemia^14^.

**Table 1:** Comparison of the main clinical and laboratory characteristics between patients with biopsy-proven renal involvement and Idiopathic hypocryoglobulinaemia vs. mixed cryoglobulinaemia.

	Idiopathic hypocryoglobulinaemia N = 9	Mixed Cryoglobulinaemia N = 16	P
HCV + N. (%)	2 (22)	16 (100)	**< 0.01**
Proteinuria > 3.5 g/day N. (%)	4 (44)	12 (75)	0.12
Haematuria N. (%)	5 (55)	11 (69)	0.5
sCr > 1.5 mg/dl N. (%)	8 (88)	15 (94)	0.6
Isolated renal involvement N. (%)	8 (88)	0 (0)	**< 0.01**
Cryoglobulinaemia N. (%)	Trace amounts of polyclonal cryoglobulins in 9 (100)	Type II in 16 (100)	**< 0.05**

Statistically significant values are in [bold].

### Renal histological features

Analysis by *light microscopy* allowed the identification of focal (3 cases) or diffuse (5 cases) membranoproliferative glomerulonephritis with duplication of glomerular basement membrane, interposition by mesangial cells, subendothelial and mesangial deposition of immune reactants, proliferation and expansion of the mesangium and intracapillary monocyte accumulation with capillary occlusion (7 of 9 biopsies), and endoluminal hyaline pseudothrombi (corresponding to hypocryoglobulin precipitates) in 6 of 9 cases. Fibrinoid necrosis was found in 2 cases. One patient had florid crescents. A mesangial proliferative pattern was detected in the remaining case. In 3 cases huge subepithelial (hump-like) deposits could be detected*.*

*Immunofluorescence staining* shows diffuse mesangial deposition mainly of IgM, IgG, and C3 and C4 also detectable in the peripheral capillary wall. Strong IgM and IgG deposition was observed in the thrombi.

**Figure 1:**
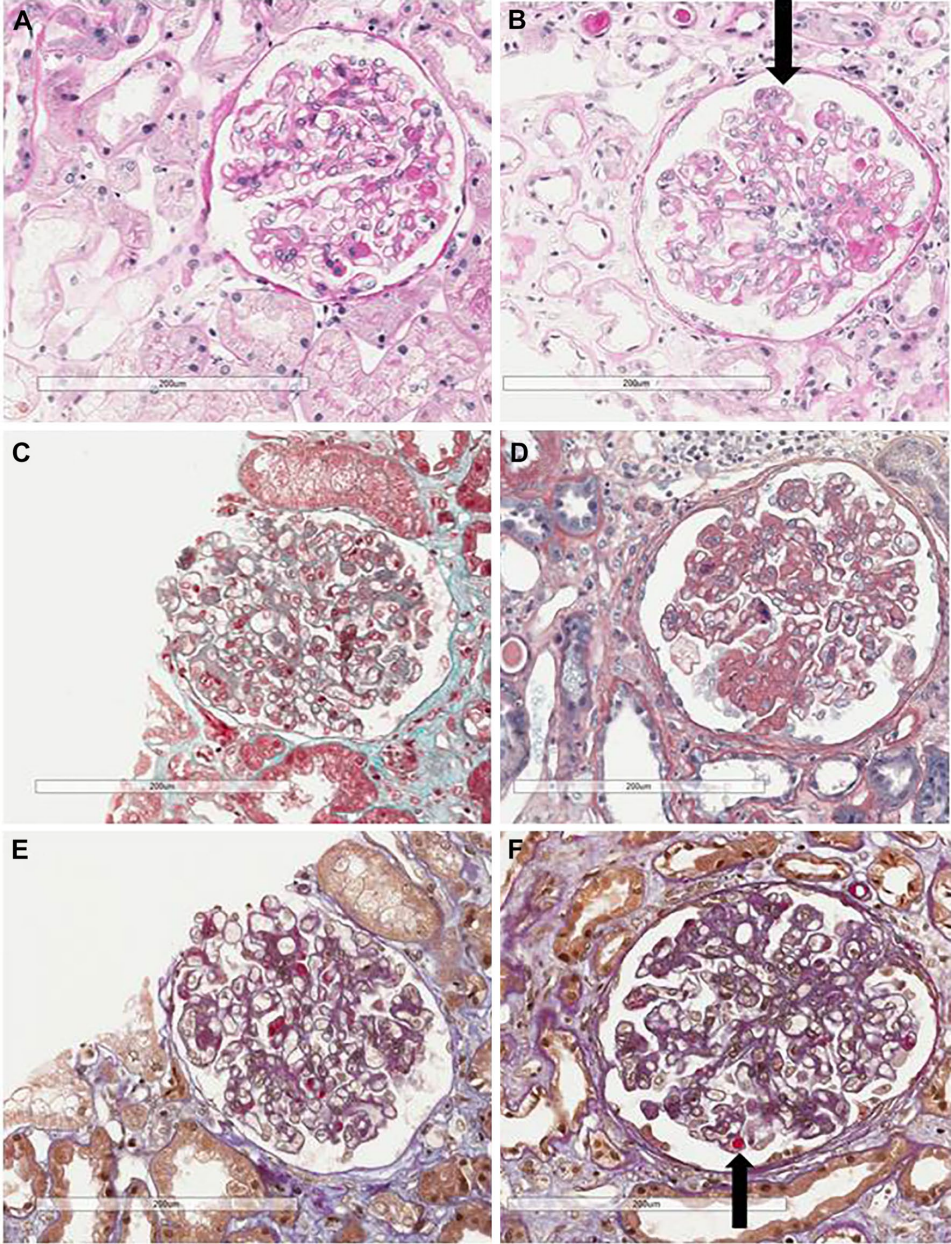
Light microscopy findings in hypocryoglobulinaemic glomerulonephritis. (**A**–**C**) PAS and Trichrome stains show mild thickening of basement membranes with segmental duplication (arrow in **B**), mild mesangial hypercellularity and increased matrix and eosinophilic refractile pseudothrombi (**A**, **B**), PAS original magnification × 200; (**C**) trichrome original magnification × 200. (**D**–**F**) Pseudothrombi appear respectively blue on PTAH stain and glassy pink (arrow in **F**) on AFOG sections (**D**, PTAH original magnification × 200; **E**, **F**, AFOG original magnification × 200).

**Figure 2:**
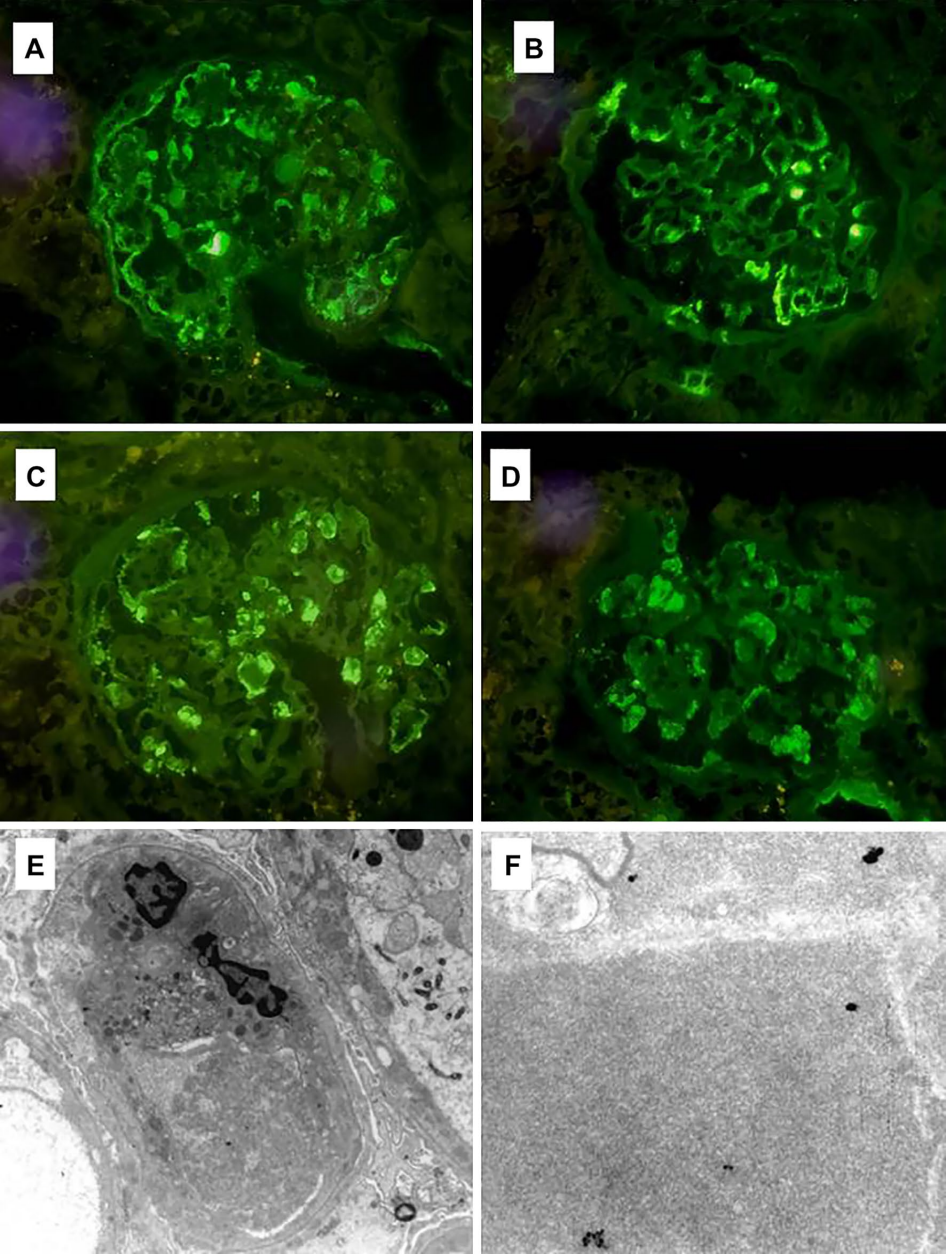
Immunofluorescence and electron microscopy features. Glomeruli show moderate granular positivity for IgM (**A**) and IgG (**B**) along glomerular capillary loops and within capillary lumina in intraluminal thrombi observed in light microscopy, with kappa (**C**) and lambda (**D**) deposits (**A**–**D**, direct immunofluorescence on fresh material, original magnification × 400). (**E**, **F**) Transmission electron microscopy, uranyl acetate and lead citrate, (**E**) X3900 and (**F**) X21000. Capillary thrombi and subendothelial electron dense deposits with vague microtubular and annular substructure. Substructure in the electron dense deposits is more noticeable at high magnification.

*Electron microscopy*, available in 7 cases, allowed detection of electron-dense deposits in the subendothelial, intramembranous and mesangial space. Deposits had a structured appearance in 5 cases and were amorphous in 2. Typical histologic features of hypocryoglobulinaemic nephritis are shown in Figs. 1 and 2.

### Treatment of patients with idiopathic hypocryoglobulinaemia

Patients were treated on the basis of the severity and the prevailing symptoms, as follows. Constitutional symptoms and arthralgias were treated with nonsteroidal anti-inflammatory drugs. Patients with skin lesions received steroids alone (12 patients), colchicine (1 patients), colchicine plus steroids (10 cases), steroids and hydroxychloroquine (3 patients), and dapsone (5 patients). Patients with peripheral neuropathy were given steroids (# 13) or Rituximab (# 6). Nine patients received symptomatic therapies (including duloxetine, pregabalin, and gabapentin). Glomerulonephritis patients were treated with steroids alone (# 3), steroids and plasmapheresis (# 1), steroids and Tacrolimus (# 1), Rituximab “4 + 2 improved protocol”^14^ (#3). One patient presenting features of crescentic nephritis was treated with an intensified B depletion protocol, consisting of a combination of Rituximab (4 + 2 scheme), two low doses of Cyclophosphamide, and 3 pulses of methylprednisolone followed by oral prednisone tapered until discontinuation in three months^15^.

The different applied therapeutic approaches mirrored the heterogeneity of the clinical presentations. Overall, constitutional symptoms disappeared or ameliorated in all the treated patients. Patients with skin manifestation achieved a complete remission within 2 months. In patients with isolated neurological manifestation, sensory symptoms disappeared or improved following treatment in 18 out of 28 patients (64%). An improvement in the clinical neuropathy disability score was also observed. Electromyography examination revealed that the amplitude of compound motor action potential had increased in 16 patients (57%). When referring to patients with kidney involvement, a complete renal response was observed within 6 months after treatment in all cases.

## Discussion

The current study focuses on the possible clinical impact of very low levels of circulating cryoglobulins (hypocryoglobulins). Patients with hypocryoglobulins might present with skin ulcers, multiple mononeuritis, glomerulonephritis together with constitutional symptoms and arthralgia. While the vast majority of patients with cryoglobulinemic vasculitis, especially those with renal involvement, have monoclonal IgM–k with polyclonal IgG (type II) cryoglobulins, high level of RF and decreased C4, and HCV infection, patients with *idiopathic hypocryoglobulinaemia* have trace amounts of polyclonal (type III) cryoglobulins. They are also negative for HCV infection in the majority of cases, and could have normal rheumatoid activity and complement levels. While it seems reasonable to suspect that hypocryoglobulins might trigger rheumatoid factor activity and levels of complement activation below the standard detection limits, physicians could face a major diagnostic challenge when handling a clinical presentation suggestive of cryoglobulinaemic vasculitis, albeit negative for HCV infection, in the absence of detectable cryoglobulins, and with normal levels of complement and rheumatoid factor. In this case, the conventional hallmarks of the diagnosis of mixed cryoglobulinaemia remain unfulfilled. A careful search for cryoprecipitable immune complexes might represent a further tool to close the serological gap for the diagnosis of the full spectrum of cryoglobulinaemia. Some technical considerations are worth emphasizing. First, the diagnostic work-up aimed at ruling out the presence of circulating cryoglobulins should require stringent controls on blood sampling and processing. Second, the significance of trace amounts of cryoglobulins is generally underestimated, and, especially in HCV-negative patients, should draw the attention of the clinicians. Third, an in-depth characterization of the manifestations is required in order to avoid misdiagnosis. This should include tissue examination especially if kidney involvement is suspected.

However, the similarities in the outcome, when compared to mixed cryoglobulinaemia, support the concept that hypocryoglobulinaemia and cryoglobulinaemia may represent two sides of the spectrum of a common pathogenic process. Rituximab has substantially changed the natural history of HCV- and non-HCV-related mixed cryoglobulinaemia^9,16^ and represents the best tool for the more severe cases of idiopathic hypocryoglobulinaemia. Rituximab is safer than conventional immunosuppressants and can achieve long-term remission, especially with intensive regimens, such as the so-called “improved protocol”^14^ and the “intensive B depletion therapy”^15^.

Our findings have the potential to redefine the clinical features and diagnostic pathways for cryoglobulinaemia, introducing the concept of idiopathic hypocryoglobulinaemia, thereby promoting more widespread recognition and earlier diagnosis. From this perspective, to rule out false positive results, histology remains diriment to confirm this new entity. More research is required to gain insight into the immunological abnormalities underlying these conditions and to develop novel screening, diagnostic, and prognostic tools. Besides, while in this study we opted for the definition of hypocroglobulinemia, future studies, including those seeking consensus among experts, are required to define the most appropriate nomenclature for patients with trace amounts of cryoglobulin. Similarly, we acknowledge that polyneuropathy can have many causes not directly related to the detected trace concentration of cryoglobulins. However, all our cases could be more appropriately defined as *multiple mononeuritis*, which is a typical feature of vasculitis of “*vasa nervorum*”.

Waiting for new data, patients who present high clinical suspicion for cryoglobulinaemic vasculitis, especially glomerulonephritis, and yet test negative for cryoglobulinaemia detectable by standard techniques, should be carefully evaluated even in the absence of HCV infection, RF activity and signs of complement consumption.

## Materials and methods

### Patients

The present cohort includes 237 patients, median age 60.8, range 22–97 years, 100 males and 137 females, having trace amounts of cryoglobulins (< 0.5% cryocrit) detected in the Laboratory of CMID, San Giovanni Bosco Hub Hospital and University of Turin between 2008 and 2021. Patients presenting with high clinical suspicion of autoimmune systemic conditions yet negative for the detection of cryoglobulinemia with the standard technique were tested for hypocryoglobulins. All patients underwent an accurate list of investigations as detailed in Table 1S (“Supplementary materials”). All patients with urinary abnormalities underwent kidney biopsy.

### Ethical approval

All subjects provided informed consent according to the Declaration of Helsinki. This study was performed according to the local rules of off-label therapy in Piedmont (Northwest Italy).

The study was approved by the Ethical Committee Board of the Azienda Ospedaliera Universitaria Città della Salute e della Scienza di Torino (Academic Hospital Città della Salute e della Scienza di Torino)—Azienda Ospedaliera Ordine Mauriziano (Ordine Mauriziano HospitaL)—Azienda Sanitaria Locale Città di Torino (City of Torino Local healthcare facilities), Torino, IT; (N. protocol 0092056 of 2020).

### Detection of hypocryoglobulins

Detection of serum hypocryoglobulins and measurement of hypocryocrit were performed by a modified precipitation technique in hypoionic medium^12^. The procedure follows in part the standardized steps of the detection of cryoglobulins. Careful quality control of sampling and transfer to the laboratory at 37 °C were assured. Blood was sampled in four pre-warmed tubes with no anticoagulant and allowed to clot at 37 °C for > 2 h. Serum was separated by centrifugation (3000 rpm) for 20 min at 37 °C and the supernatants were collected in Wintrobe tubes and then diluted with an equal volume of bidistilled water (50% serum and 50% distilled water). Diluted sera were incubated at 4 °C for 7–10 days. The precipitates were then separated by centrifugation (one 15 min cycle at 3000 rpm) at 4 °C. Supernatants were then removed from the Wintrobe tubes, leaving the sediment at the bottom. An alkaline wash solution (A) (Helena Kit, Bioscience Europe) was added in order to reach 50 mm of the first graduated tube. Sediment was resuspended for every sample and suspension was centrifuged at 4 °C for 20 min at 3000 rpm. The supernatant was then discharged, and this step was repeated twice. After washing, the sediment was mixed with 200 microL of a cryoglobulin solubilizing solution containing BSA and peptide-like compound, B solution of the kit, and 50 microL of physiologic solution. Purified, re-dissolved precipitates were analysed one to two days after purification and the subsequent procedures were performed at room temperature. Typing of the cryoprecipitate was assessed by agarose gel electrophoresis and immunofixation using the SAS-1 machine (Helena Biosciences Europe, Tyne and Wear, United Kingdom). The clinical accuracy of the methods was evaluated in a cohort of 100 healthy donors. None of them had levels of hypocryoglobulins detected.

**Figure 3:**
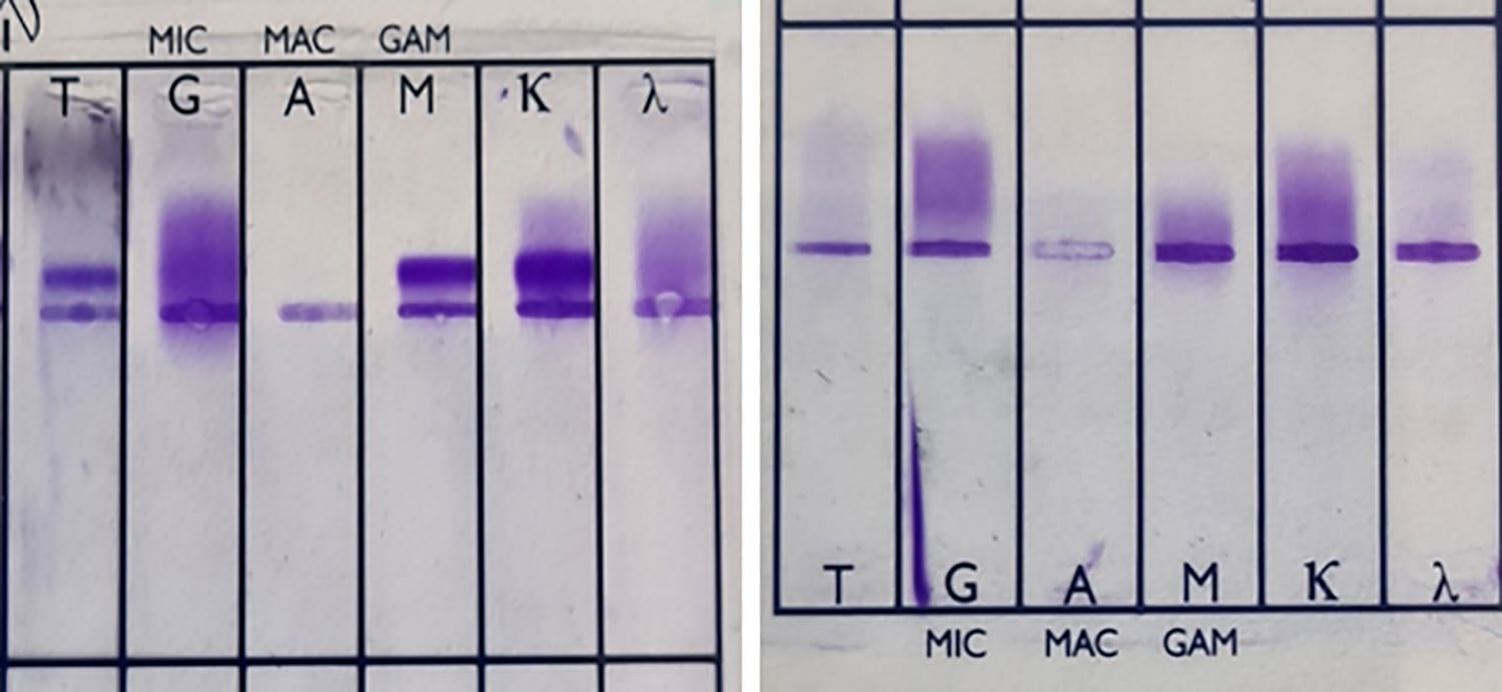
Patterns of immunoglobulin identification of the hypocryoglobulimemic precipitates by gel electrophoresis and immunofixation. Left side: example of type II hypocryoglobulin with policlonal IgG and monoclonal IgM-k. Right side: example of type III hypocryoglobulin with policlonal IgG and policlonal IgM. The grouping of gels different parts of from different gels. Full-length gels are included in a “Supplementary Information” file.

Examples of patterns of immunoglobulin identification of the hypocryoglobulinemic precipitates by gel electrophoresis and immunofixation are reported in Fig. 3.

### Statistical analysis

All collected data were first reported in a descriptive manner, then grouped according to the historical clinical manifestations and HCV status. Quantitative data were described as median and range. Qualitative data were categorized into frequency and percentages and binomial confidence intervals. Due to the nature of this study, mainly descriptive statistics have been considered. When needed, non-parametric tests were used. Fisher’s exact test was used for qualitative data analysis. P < 0.05 was considered to be statistically significant. All statistical analyses were performed using SPSS version 19.0 (IBM, Armonk, NY, USA).

## Supplementary Information

Supplementary Information 1.

Supplementary Table 1.
