# Supplementary figures and images for: Recognizing the new disorder “idiopathic hypocryoglobulinaemia” in patients with previously unidentified clinical conditions
Source: Sci Rep. 2022 Sep 1;12:14904. doi: 10.1038/s41598-022-18427-x (PMC9437023; doi:10.1038/s41598-022-18427-x)

\_\_\_\_\_

\_\_\_\_\_

SAS IFE-

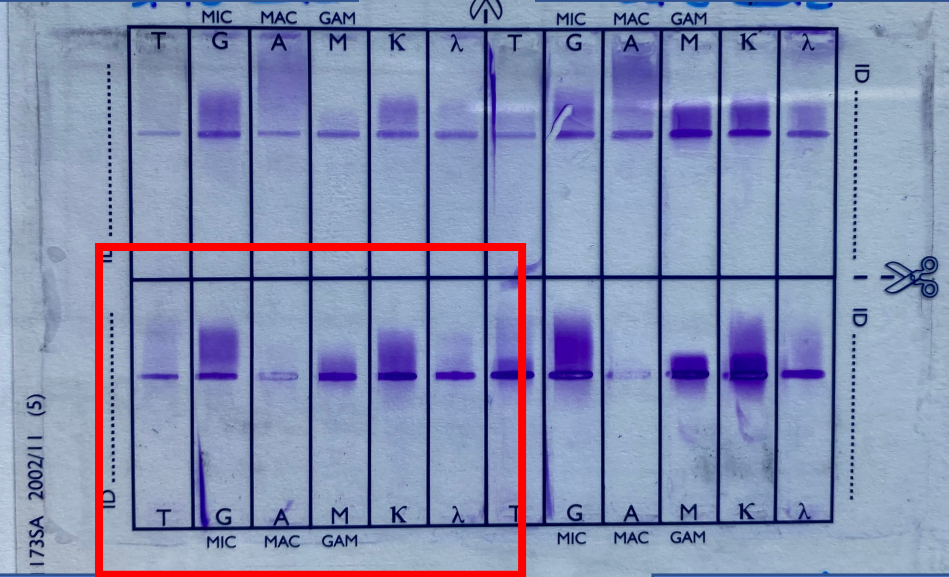

helenal BioSciences Europe

Supplement: Supplementary file 1 — Supplementary Information 1. [file 41598_2022_18427_MOESM1_ESM.pdf]
