# Supplementary material for: Recognizing the new disorder “idiopathic hypocryoglobulinaemia” in patients with previously unidentified clinical conditions
Source: Sci Rep. 2022 Sep 1;12:14904. doi: 10.1038/s41598-022-18427-x (PMC9437023; doi:10.1038/s41598-022-18427-x)
Supplement: Supplementary file 2 — Supplementary Table 1. [file 41598_2022_18427_MOESM2_ESM.docx]

Table 1S. Investigations protocol

| Accurate Anamnestic Collections | Family history, Past medical history, lifestyle, smoking habit, previous pregnancy history. |
| --- | --- |
| Immunological symptoms | Dry mouth, dry eyes, photosensitivity, mouth ulcers, skin rash, recurrent infections, Raynaud's phenomenon, previous thrombosis. |
| FBC and Coagulation profile | Blood smear if FBC abnormalities were identified. |
| Serum chemistries | Liver and kidney chemistries, LDH, glycemic profile, thyroids and hormonal profiles. |
| Urinalysis and Urine sediment | Performed by specifically trained nephrologists |
| 24-h urine collections |  |
| Ig and Complement levels | IgG/A/M; C3, C4, Coombs tests. |
| Rheumatoid factor |  |
| Viral serologies | HCV, HBV, HIV, and Epstein-Barr virus |
| Acute phase reactant | ESR, CRP, protidogram |
| Autoantibodies | Antinuclear antibody; double-stranded DNA, anti-Sm, Ro/SSA, La/SSB, and U1 ribonucleoprotein, anti Scl-70, antibodies to citrullinated peptide antigens, antineutrophil cytoplasmic antibody, antiphospholipid antibodies. |
| Biopsy | Skin, Kidney, Bone Marrow, lymphnodes (according to clinical presentation) |
| Electromyography | 4 limbs |
| Imaging | Imaging studies were guided by the clinical presentation (E.g., computed tomography scan of the chest, abdomen, and pelvis when searching for a lymphoproliferative disorders) |
| Arthralgia | X-rays and ultrasound investigations including power doppler |
| Nail video-capillaroscopy |  |
